# Successful Aging among Elders Living in the Mani Continental Region vs. Insular Areas of the Mediterranean: the MEDIS Study

**DOI:** 10.14336/AD.2015.1002

**Published:** 2016-05-27

**Authors:** Anargiros Mariolis, Alexandra Foscolou, Stefanos Tyrovolas, Suzanne Piscopo, Giuseppe Valacchi, Nikos Tsakountakis, Akis Zeimbekis, Vassiliki Bountziouka, Efthimios Gotsis, George Metallinos, Dimitra Tyrovola, Josep-Antoni Tur, Antonia-Leda Matalas, Christos Lionis, Evangelos Polychronopoulos, Demosthenes Panagiotakos, for the MEDIS study group

**Affiliations:** ^1^Health Center of Areopolis, General Hospital of Sparta, Areopolis, Greece; ^2^Department of Nutrition and Dietetics, School of Health Science and Education, Harokopio University, Athens, Greece; ^3^Parc Sanitari Sant Joan de Déu, Fundació Sant Joan de Déu, CIBERSAM, Universitat de Barcelona, Barcelona, Spain; ^4^University of Malta, Nutrition, Family and Consumer Studies Office, Msida, Republic of Malta; ^5^Department of Life Sciences and Biotechnology, University of Ferrara, Ferrara, Italy; ^6^Clinic of Social and Family Medicine, School of Medicine, University of Crete, Heraklion, Greece; ^7^Health Center of Kalloni, General Hospital of Mitilini, Mitilini, Greece; ^8^Research Group on Community Nutrition and Oxidative Stress, Universitat de les Illes Balears & CIBERobn, E-07122 Palma de Mallorca, Spain

**Keywords:** successful aging, longevity, Mani region, Mediterranean islands, cardiometabolic risk, lifestyle

## Abstract

To evaluate the role of geography i.e., continental vs. insular Mediterranean, on successful aging among older inhabitants. During 2005-2014, 2693 elderly (aged 65 to 100 years) individuals from 21 Mediterranean islands in Greece, Italy and Spain as well as Cyprus, Malta, and the rural region of Mani (southeast continental region of Greece keeping old-time traditions), were voluntarily recruited. Successful aging was evaluated using a validated index composed of 10 health-related socio-lifestyle and clinical characteristics. After accounting for age, sex, body mass index (BMI), physical activity, smoking habits, MedDietScore and access to health care services, the older inhabitants of islands were found to have a higher level of the successful aging index when compared to their counterparts in Mani (Beta=0.174, p<0.001); moreover, islanders exhibited slightly more years of “good” health (68.7 vs 68.4 years for Mani residents (p=0.99)). However, compared to the residents of Mani, islanders had 1.64 times higher odds (95%CI, 1.08-2.48) for having hypertension, 2.4-times higher odds (95%CI, 1.34-4.21) for having diabetes and 1.52 times higher odds (95%CI, 0.97-2.38) for having hypercholesterolemia. Engaging in physical activities and healthy dietary habits were the major determinants of healthy aging, among islanders as compared to their counterparts of continental Mani region. Elder residents of the continental Mani area enjoyed a better health status, whereas elder islanders had a higher level of successful aging; a finding which could be attributed to differences in lifestyle among elders.

It is true that the aged population is increasing in all over the world and the percentage of 60-year-old people follows the same trend. In 2013 this aging group comprised 841 million people and it is expected to have surpassed the number of 2 billion by the year 2050 [1]. Thus, it is considered necessary to carry out a research on how this target group could have successful aging. Longevity could be the result of genetics, but also of lifestyle factors, such as physical exercise and healthy diet. Probably the place of living along with the social life are also significant ones. Longevity and successful aging are two difficult senses as they rely on several factors, which may be still completely unidentified till nowadays. Nevertheless, the successful aging and the research that it is carried out in this sector, refers to the issues relevant to the public health because if these senses are clarified the global population will be benefited. The general sense about successful aging is focused on five pillars according to a study, which was conducted in 2015. Euphoria, continuous activity, educational background, financial stability and health are the issues that are regarded as the main components of successful aging, with euphoria as the most important of them [2]. Rowe and Kahn, in 1997, were two of the first researchers of successful aging, who evinced that successful aging is based on several factors but they focused only on three of them. The first factor is the avoidance of disease and disability, the second is the maintenance of high standards of physical and mental operation and the last is the continuous commitment in social and productive activities [3]. From observations carried out mostly with people of Ikaria, an island with population having the least health problems, the secret to longevity and the successful aging lies mainly on the following points: stocking up on herbal, the adoption of a Mediterranean type diet, mimic mountain living, fasting occasionally, taking regular naps and focusing on family and friends bonds as their first priority [4]. Moreover, the term “active aging” could be included in the general sense of the term “healthy” or “successful aging”. The “active aging” is related not only to physical but also to the mental health over the years through a more general change in the lifestyle [5]. Nevertheless, the vulnerability and the complexity of the aged people’s health is standardized, whereas a lot of the aged are frail. So, in the whole effort for the amendment of those people’s health, with restricted sources, public health should be focused on the know-how, which needs to be implemented, so as to deal with all the complex incidents and the factors, which form the health of the aged [6]. Yet, besides all the above, the alimentation factor is what is directly linked to the quality of living. Indeed, a form of alimentation based on the models of the Mediterranean Diet, has proved to contribute to the adoption of a specific way of living and a tradition, which, in their turn, contribute to a better standard of health and quality of life. According to a study in Italy among people, who were city or province dwellers, the rate of the Mediterranean Diet adoption differed a lot. City inhabitants were far away from the models of that diet unlike the inhabitants of the provinces, who were very close to it. Longevity and successful aging were also detected because of that diet. People who lived in the countryside had not only a bigger life expectancy but also better living conditions [7]. However, it seemed that despite the factor of nutrition quality which each aged person followed, a great percentage of those, who reside on mountainous regions suffer from metabolic syndrome and this happens because the access to health care is restricted [8].

Except for that, people, who lived off cities and had personal means of transport such as their own car or a relative of theirs to give them a lift, had greater and more frequent access to health care services in relation to those, who hadn’t got the same amenities [9]. Moreover, on islands, where the health care services provided nutritional services as well, it seemed that the islanders had better results during the third age, since incidents of diabetes or hypercholesterolemia, cardiovascular diseases (CVD) and obesity, were clearly reduced [10] proving that in this way nutrition is the factor, which can secure the “healthy aging”. Certainly, as it was proved from a research carried out in Portugal, the aged couples do not pay particular attention to their nutrition standards whereas the single aged persons, who live on their own, seem to have higher score on Health Diet Indicator. Moreover the lower educational standard of the household head together with the house location in less urban areas, are those which state the healthy nutrition adoption [11] and therefore these are the people who have healthier aging.

Given the lack of current data regarding successful aging in combination with the accessibility and visitability at health services by those who live in insular regions, where health care services are poorer than those who live in continental regions of Greece, it is proved that the latters have certainly better health care services although the geographic and cultural profiles are similar to those of the formers. Besides, there are incomplete comparison elements between these two groups concerning the cardiometabolic factors and the comparison referring to the accessibility to health care services. The aim of the present work was to evaluate the role of the area of living, and particularly a continental region in the southeast part of Europe, i.e., Mani, Greece versus insular Mediterranean areas (i.e., Aegean islands, Crete, Ionian islands, Cyprus, Malta, Sardinia, Corsica), on cardiometabolic risk and successful aging, among older adults (>65 years of age). It is notable that Mani and the Mediterranean islands have common cultural characteristics and during the past centuries, they had developed extensive trading, sharing goods and local products, as well as various cultural and behavioral particularities.

## MATERIALS AND METHODS

### Methodology

Mediterranean Islands (MEDIS) study is an ongoing, large-scale, multinational project in the Mediterranean region, supported by the Harokopio University and the Hellenic Heart Foundation, which aims to explore the association of lifestyle habits, psycho-social characteristics and living environment, on cardiometabolic factors, among older people (>65 years), permanent residents of the Mediterranean area.

### The MEDIS Study’s sample

During 2005-2014, a population-based, multi-stage convenience sampling method was used to voluntarily enroll elders from the 21 Mediterranean islands: Malta Republic (n=250), Sardinia (n=60) and Sicily (n=50) in Italy, Mallorca and Menorca (n=111), Republic of Cyprus (n=300) and the Greek islands of Mitilini (n=142), Samothraki (n=100), Cephalonia (n=115), Crete (n=131), Corfu (n=149), Limnos (n=150), Ikaria (n=76), Syros (n=151), Naxos (n=145), Zakynthos (n=103) and Salamina (n=147), Kassos (n=52), Rhodes and Karpathos (n=149), Tinos (n=129), as well as the rural region of east Mani (n=295, 157 men aged 75±7 years and 138 women aged 74±7 years) (a Greek peninsula, which is the southest, continental area of Europe, with a total population of 13,005 people (census 2011), has morphological and cultural specificities, which are not come across in the rest of Greece. It is an infertile and rocky peninsula, of which the linkage to ancient Sparta bequeaths to its residents -mainly self-employed fishermen and farmers, living in small villages, and keeping on a lot of their activities) the traditional way of living of the past. Their nutrition is austere and frugal, based on the products of their land with the olive oil gaining an eminent position) (https://en.wikipedia.org/wiki/Mani_Peninsula), were included. According to the design of the study, individuals who resided in assisted-living centers, had a clinical history of cardiovascular disease (CVD) or cancer, or had left the island for a considerable period of time during their life (i.e., >5 years) were not included in the study; these exclusion criteria were applied because the study aimed to assess lifestyle patterns that were not subject to modifications due to existing chronic health care conditions or by environmental factors, other than living milieu. A group of health scientists (physicians, dietitians, public health nutritionists and nurses) with experience in field investigation collected all the required information using a quantitative questionnaire and standard procedures. Thus, for the present work information from 1,344 men, aged 75±8 years and 1,349 women, aged 74±7 years was analysed; stratified into two main groups, i.e. people from the continental region of Mani, and the rest of MEDIS’ study participants.

### Bioethics

The study followed the ethical considerations provided by the World Medical Association (52^nd^ WMA General Assembly, Edinburgh, Scotland; October 2000). The Institutional Ethics Board of Harokopio University approved the study design (16/19-12-2006). Participants were informed about the aims and procedures of the study and gave their consent prior to being interviewed.

### Evaluation of clinical characteristics

All of the measurements taken in the different study centers were standardized and the questionnaires were translated into all of the cohorts’ languages following the World Health Organization (WHO) translation guidelines for tools assessment (www.who.int/substance_abuse/research_tools/translation/en/).

Weight and height were measured using standard procedures to attain body mass index (BMI) scores (kg/m^2^). Overweight was defined as BMI between 25 and 29.9 Kg/m^2^, while obesity was defined as BMI >29.9 Kg/m^2^. Moreover, waist circumference (in cm) was measured in the midpoint between the 12^th^ rib and the iliac crest and hip circumference (in cm) was measured around the buttocks. Central fat was defined as waist circumference greater than 102 cm for men and 88 cm for women. Diabetes mellitus (type 2) was determined by fasting plasma glucose tests and was analyzed in accordance with the American Diabetes Association diagnostic criteria (glycated haemoglobin A1C>6.5 or fasting blood glucose levels greater than 126 mg/dl or 2-h plasma glucose > 200 mg/dl during an oral glucose tolerance test-OGTT- or a random plasma glucose > 200 mg/dl or they have been already diagnosed with diabetes). Participants who had blood pressure levels >140/90 mmHg or used antihypertensive medications were classified as hypertensive. Fasting blood lipids levels (HDL-, LDL-cholesterol and triglycerides) were also recorded and hypercholesterolemia was defined as total serum cholesterol levels >200 mg/dL or the use of lipid-lowering agents according to the NCEP ATPIII guidelines [12]. The coefficient of variation for the blood measurements was less than 5%.

### Evaluation of lifestyle and socio-demographic characteristics

Dietary habits were assessed through a semi-quantitative, validated and reproducible food-frequency questionnaire [13]. To evaluate the level of adherence to the Mediterranean diet, the MedDietScore (possible range 0-55) was used [14]. Higher values for this diet score indicate greater adherence to the Mediterranean diet. Participants were encouraged to report on the history of their diet (i.e. number of years this dietary pattern had been in place). Basic socio-demographic characteristics such as age, sex, retired or still at work, adequate annual income (defined as >9.000€ per person), as well as lifestyle characteristics, such as smoking habits and physical activity status, data on frequency of outdoor activities for pleasure and fun, as well as, participation in social events (per week), were also recorded. More particularly, current smokers were defined as smokers at the time of the interview. Former smokers were defined as those who previously smoked, but had not done so for a year or more. Current and former smokers were defined as ever smokers. The remaining participants were defined as occasional or non-smokers. Physical activity was evaluated in MET-minutes per week, using the shortened, translated and validated into Greek, version of the self-reported International Physical Activity Questionnaire (IPAQ) [15]. Frequency (times per week), duration (minutes per session) and intensity of physical activity during sports, occupation and/or leisure activities were assessed. Participants were instructed to report only episodes of activity lasting at least 10 minutes, since this is the minimum required to achieve health benefits. Physically active were defined those who reported at least 3 MET-minutes.

Symptoms of depression during the previous month were assessed using the validated Greek version (also translated in all the cohort’s languages) of the shortened, self-report Geriatric Depression Scale (GDS) (range 0-20) [16].

A successful aging index (SAI) ranging from 0 to 10 has been previously developed and tested, using 10 attributes, i.e., education, financial status, physical activity, body mass index, depression, participation in social activities with friends and family, number of yearly excursions, total number of clinical CVD risk factors (i.e., history of hypertension, diabetes, hypercholesterolemia) and level of adherence to the Mediterranean diet through the MedDietScore [17]. Years of “good” health were measured as the age of the participants that did not seek for medical assistance due to co-morbidities.

Presence of small physicians’ offices, healthcare centers, or hospitals, in the area of living, as well as the annual number of visits to healthcare centers, etc, for regular check-up of the health status by the participants were also recorded.

Further details about the MEDIS study protocol have been extensively been published elsewhere [17, 18].

### Statistical analysis

Binary logistic regression models were used to evaluate the association between participants’ characteristics (i.e., age, sex, area of residence, physical activity, adherence to the Mediterranean diet, smoking habits, access to health care services) and presence of cardiometabolic factors (hypertension, diabetes mellitus, hypercholesterolemia, obesity). Results are expressed as odds ratios and the 95% confidence intervals. Linear regression models were used to evaluate the association between participants’ characteristics (i.e., age, sex, area of residence, physical activity, adherence to the Mediterranean diet, smoking habits, access to health care services) and successful aging index. Linear regression model was also used to examine the relationship between access to health care services during winter or summer and successful aging. Results are expressed as standardized beta coefficients, in order to allow comparisons between variables. Normality was tested using P-P plots. Independence between categorical variables was tested using the chi-square criterion. The significance level was set up at 0.05. SPSS software (version 20) was used for all calculations (SPSS Inc., Chicago, Il, USA).

## RESULTS

In Table 1 basic socio-demographic, lifestyle and clinical characteristics of the participants are presented. Islanders, were less likely to be retired, as approximately 24% of them reported to be still working, but were more likely to engage in physical activities and showed better adherence to the traditional Mediterranean diet. Although levels of obesity were more or less, similar, central obesity was much more prevalent among older adults in the Mani region, whereas, history of the common cardiometabolic factors (hypertension, diabetes and hyper-cholesterolemia) was higher among islanders. Moreover, islanders had higher depression scores, when compared to their counterparts from continental Mani region.

### Area of living and Cardiometabolic risk

Cardiometabolic risk was indirectly measured as the sum of four common cardiovascular disease risk factors, hypertension, diabetes, dyslipidemia and obesity. Islanders had higher score as compared to those living in Mani region (1.7±1.1 vs. 1.3±0.9 risk factors / participant, p<0.001). Adjusting only for age and sex, those living in the islands had 1.50 times higher odds (95% CI, 1.17 - 1.90) for having hypertension than those living in Mani, 1.49 times higher odds (95% CI, 1.08 - 2.05) for having diabetes mellitus, 1.77 times higher odds (95% CI, 1.37 - 2.29) for having hypercholesterolemia and 1.30 times higher odds (95% CI, 0.99 - 1.73) for being obese. After further adjustment for potential confound factors, i.e., physical activity, smoking habits and MedDietScore, islanders had still 1.50 times higher odds (95% CI, 1.03 - 2.17) for having hypertension, 2.13 times higher odds (95% CI, 1.39 - 3.91) for having diabetes mellitus, and 1.83 times higher odds (95% CI, 1.23 - 2.72) for having hypercholesterolemia as compared to those living in Mani region (p=0.0001).

**Table 1:** Lifestyle, psychosocial and clinical characteristics of the MEDIS study participants.

	Mediterranean Islands (n=2,399)	Mani continental region (n=295)	*p*
Age (years)	74±8	75±7	0.30
Men, %	49.5	53.2	0.23
Retired, %	76.7	98.0	0.001
Basic school (up to 6 years)	66.1	51.7	<0.001
Adequate financial status, %	18.6	25.4	0.05
Outdoor activities (t/wk)	2.4±1.4	1.5±1.1	<0.001
Social events (t/wk)	1.8±0.9	1.3±0.9	<0.001
Participation in social networks, %	38	14	<0.001
Living alone, %	30	25	0.24
Physical activity, %	44.5	13.9	<0.001
Smoking habit, %	35.1	37.3	0.45
MedDietScore (range 0-55)	36±5	32±4	<0.001
Body Mass Index (Kg/m^2^)	28.3±4.7	27.8±4.1	0.05
Obesity, %	32.2	26.1	0.03
Central fat, %	75.5	86.0	0.001
History of hypertension, %	63.5	53.9	0.001
History of diabetes, %	23.1	16.8	0.02
History of hypercholesterolemia, %	49.7	35.6	0.001
Adherence to medication, %	92	55	<0.001
Geriatric Depression Scale (range 0-20)	4.1±3.6	2.4±3.3	<0.001
Successful Aging Index (range 0-10)	1.55±0.88	1.40±0.63	<0.001
Access to health care services (visits/year)	2.6±1.2	1.1±0.4	<0.001

When “*access to health care services*”, as measured by the accessibility to the centers, was taken into account in the models, islanders had 1.64 times higher odds (95% CI, 1.08 - 2.48) for having hypertension than elders who lived in the Mani region; as far as diabetes mellitus is concerned, islanders were still associated with 2.4 times higher odds (95% CI, 1.34 - 4.21) for having this disease, and the odds ratio for having hypercholesterolemia was 1.52 times (95% CI, 0.97 - 2.38) higher in islanders than of those who live in Mani.

### Area of living and Successful aging

It is notable that the level of SAI was low in both islanders and Mani region participants. When islanders and people who lived in the Mani region were contrasted, the level of successful aging among the former was found to be higher by 11% compared to the latter (1.55±0.88 vs. 1.40±0.63, p<0.001); moreover, men had higher SAI than women (1.67±0.82 vs. 1.42±0.78, p<0.001), whereas, a significant, inverse correlation was observed between participants’ age and SAI (r=-0.12, p<0.001). After adjusting only for age and sex, living in the Mediterranean islands was associated with better SAI, as opposed to continental Mani region (Beta=0.048, p=0.01). Further adjustments for physical activity status, body mass index, dietary habits through the MedDietScore, and smoking habits, confirmed the previous association of better successful aging among islanders, with even stronger significance (Beta = 0.08, p=0.003). When the variable “access to health care system” (as measured by the accessibility) was taken into account, the effect of living in an island as opposed to a continental region, was even much stronger (Beta = 0.20, p<0.001). Being engaged in physical activities was the primary determinant factor making those who live in the Mediterranean islands having better successful aging index (Beta=0.158, p<0.001), following by the level of adherence to the Mediterranean diet -as measured by the MedDietScore- (Beta=0.104, p<0.001), participation in outdoor activities (Beta=0.209, p<0.001), participation in social events (Beta=0.370, p<0.001) and participation in social networks (Beta=0.099, p<0.001). Regarding the other factors, aging was a major determinant of successful aging - as expected-, since for each year that goes by, a reduction of 0.147 units in the SAI was observed (p<0.001).

Adjusting for times/year/health center in winter, summer and access to health care services, if someone visits a health center during the winter, has an increase of 0.19 units in successful aging index but with of low statistical significance (p=0.23), contrary to those who visit it during summer having a reduction of 0.42 units in successful aging index (p<0.05). Besides, those who have access to health care services have a reduction of 0.25 units in successful aging index (p<0.01) (Table 2).

**Table 2:** Results from linear regression models that evaluated the association between area of living (islanders vs. Mani continental region) (independent variable) and successful aging index, SAI (outcome), among MEDIS study participants.

	Model 1	Model 2
Islanders vs. Mani continental residents	0.05 (p=0.01)	0.17 (p<0.001)
Age (per 1 year)	-0.13 (p<0.001)	-0.15 (p<0.001)
Men vs. women	0.16 (p<0.001)	0.12 (p<0.001)
Body Mass Index (kg/m^2^)	-	-0.06 (p=0.05)
Physical Activity (yes vs. no)	-	0.16 (p<0.001)
Smoking habits (yes vs. no)	-	-0.03 (p>0.05)
MedDietScore (per 1/55 unit)	-	0.100 (p<0.001)
Access to health care services (yes vs. no)	-	-0.100 (p<0.001)

Results are presented as standardized Beta coefficients.

## DISCUSSION

The present work revealed that Mani residents, unlike Mediterranean islanders, are of better health regarding cardiometabolic risk factors. Moreover, residents of Mani showed higher rates of compliance to pharmaceutical treatment, in comparison to the islanders, a fact that indicates both people’s awareness and higher standard of the provided health care. On the other hand, islander participants showed better quality of life, as this was expressed by their higher rates in successful aging index. This was mainly attributed to lifestyle differences, since islanders seemed to be more physically active and following a better diet, as well as to social behaviors, since islanders as compared to the Mani region inhabitants, spent more time on outdoor activities or socializing in various traditional social events. Despite the limitations due to the cross sectional nature of the study, the results presented here underline the importance of lifestyle and socialization in successful aging.

A lot of researchers attribute people’s successful aging to the public healthcare facilities provided. Better access to hospital or health centers, regular medical follow-ups, frequency in the presence of physicians and medical assistants and other healthcare providers per capita, may play a critical role in having better health, but this work revealed that this is not enough. Although islanders had lower accessibility to health care services due to the particularities of their regions, they were found to have a higher index of successful aging when compared to their counterparts of the Mani region. These rates accrued from the increased levels of obesity and sedentary life of Mani residents as yet from the reduced sociability level and companionship. On the other hand, the islanders, seemed to be closer to the traditional Mediterranean diet, controllably restricted energy intake, increased physical activities and more frequent socializing within their communities. In accordance with the article of Panagiotakos et al., relevant to the proven longevity of people of Ikaria, an Aegean Sea island, the reason which makes its residents, and generally the islanders, live more - almost 8 years- is apart from their carefree way of living, the combination of a healthy diet, i.e. the Mediterranean one, and the daily socializing [4]. In other words, though the islanders present a worse profile of risk factors they have better successful aging than their counterparts of the continental regions, who are less exposed to health risks due to health care services and their access to them. This is owed, as it has proved, not only to the nutrition but also to the physical activity and sociability of the islanders. Additionally, according to Oikonomou and Tountas, health care in Greece is generally poor in structure and it is stressed that easy accessibility to health care services does not apply to everyone due to different geographical factors. Yet, people living in rural areas are more vulnerable in this aspect [19]. This happens partly, because islanders due to their cares in combination with problematic transportation means, have more physical activity than people who live in the continental parts of Greece, where hospitalization and generally health care services are more convenient. The islanders, in their daily routine, adopt the Mediterranean diet in high rates, which has proved beneficial not only in cardiometabolic factors but also in various valuable ingredients for the psychic and mental health of the elderly people [20, 21]. Judging by a previous study in the same geographical region, it was shown that the adoption of the Mediterranean diet has a reverse relation to the arterial pressure and that a low consumption of alcoholic drinks (~ 0 - 1 glass daily) contributes to this reduction of the arterial pressure [22]. According to Mendis et al., the most crucial risk factors for cardiovascular diseases are based on the unhealthy diet, physical inactivity, smoking and the excessive alcohol consumption [23]. Contrary to that, there are some epidemiologic studies together with the instructions for the Mediterranean diet, which recommend alcohol intake and particularly that of red wine, in small quantities of 2-3 wine glasses per day along with meals, because its action has been proved beneficial in cases of cardiovascular diseases [24]. The “French paradox”, according to which, though the saturated fatty acids consumption is high, mortality due to coronary heart disease (CHD) is low, it was seen from its early stages to come in accordance with the beneficial action of alcohol intake, since there was always parallel wine consumption [25]. Yet, it is worth mentioning that alcohol consumption has binary influence depending on the consumed quantity. It has also been seen that those who consume a medium quantity appear to have a better cardiometabolic profile compared to those who consume excessive quantities or totally abstain from it [26].

Another cross-sectional study in Japan, which examined the effects of residential remoteness and other lifestyle associations concerning the prevalence of high blood pressure, showed that the relation of the appearance of the arterial pressure is directly linked to the distance between the residences and the dairy product market, enforcing in this way the belief that what counts is “where people live” [27]. As the prevalence of obesity has almost doubled over the last 3 decades, the mapping of obesity showed very bad results in the Mediterranean area and mostly in the Eastern Mediterranean. More than 50% of women in these regions were overweight and it is said that in financially weak countries or even regions, obesity is more commonly spotted [28]. Previous results of MEDIS study, revealed a strong correlation between the adoption of the Mediterranean Diet and generally a healthy lifestyle, and the developing of cardiometabolic disorders [29].

Referring to the successful aging, as it is a multifactorial parameter and not only medical, the present study showed that despite the fact that healthcare centers are not accessible to people who live on the islands, these are the ones who present better standards of living in comparison with the continental ones. The fact that the islanders do not visit health centers regularly, is not owing only to the difficulty in the access or the lack of medical provisions, but because they do not feel the need to proceed. It is also important the different approach to life which islanders possess. The slow life rhythm, the better sociability standards of the elderly, due to their non-familiarization with technology and the social network media (owing to the distance from the big urban centers where there are products and services for the education on technology) have helped them to seek different pastimes. They are used to busing themselves mostly with nature and people, the carefree life, the obligatory sometimes natural activity and isolation due to insularity and at times disruption of the communication means, owing to bad weather conditions and healthy nutrition as well. Islanders seem to have better SAI and longevity because of this, no matter how healthy they are pathologically. In other words, their health does not present problems such as those that require consultation by medical personnel. Instead, the residents of the continental areas, who undoubtedly have access to bigger, better and more efficiently organized in personnel and technology health centers or hospitals due to road linkage to towns, big cities and the capital city, proceed more often requiring medical consults or hospital care because their bodily or psychological health conditions may be full of problems, which cannot be coped with at home. Thus, the “successful aging” is relevant to several risk or causality factors. These factors are mainly biological, medical, environmental or simply based on lifestyle, which, in their turn, have each one, their own gravity. For example, it is not necessary for every person to get sick, but it is almost certain that they will have their immune system influenced sometime during their lifetime. Thus, as it was mentioned above the disease absence as risk factor, Stordal et al. make clear the fact that it is not only linked to mental or physical disease but also with the whenever existing risk factor for the respective disease [30]. Yet, Franzon et al. with a prospective study in Sweden, considering the relations among some modifiable factors, such as that of middle age, aging and physical or mental function in relation to the survival at a very old age, showed that a normal BMI during the middle age and the abstention from smoking, related to successful aging and longevity, setting smoking and bodily weight in the crucial risk factors [31]. Moreover, another study in Taiwan includes, in the risk factors for “successful aging”, the age, the educational level, the depression symptoms, the cognitive functions and the ability of applying of medical tests, suggesting that multiple trajectories of multi-morbidity are signs of health surcharging together with risks for “successful aging” [32].

It is therefore recommended that another factor should be searched, possibly the adoption of the Mediterranean Diet, the increase in physical activity, giving up smoking as the islanders do, in combination with their positive attitude to life due to the nice climate and their touch with the sea, which justifies better health condition and therefore not need for visiting health care services.

There was some difference spotted in the present research in Mani region. Mani residents, according to the indexes, are in better health condition than the islanders or at least the islanders have more chances for acquiring high blood pressure, diabetes type 2, hypercholesterolemia and obesity, than the Mani peninsula residents. This possibly happens because that mentioned area as peninsula resembles the environment of an island in combination with better medical care due to easier access to the rest continental country. For this frame of mapping of the health and the dietary habits of the insular population of the Mediterranean there was born the idea of relative recording of the elements collected in comparison with those of a distinguished population such as that of Mani, who have common roots with the insular mentality. In case of Malta, there is such healthcare system that allows all citizens, no matter how urbanized they are or not, to have access to health care services, which is satisfactorily funded and it is absolutely properly organized. However, Maltese, though characterized by high life expectancy rate, in comparison to other European countries, are prone to obesity whereas the non-contagious diseases create serious anxiety. For that reason it has been supported that investment strategies are demanded so that residents’ care should be displaced off hospitals apart from the regularity [33]. Yet, in Cyprus, another Mediterranean island, since long time ago integrated in MEDIS study, though the state health system bears serious problems, mainly due to recent financial crisis, which focus on long waiting lists and lack of medical technologies, in other words residents’ access to health care services is malfunctioning, Cypriots on the whole enjoy satisfactory standard of health in comparison to other countries [34].

### Limitations

This is a cross-sectional survey, and therefore lacks of causal relationships. Estimation of successful aging among elders is a difficult task, because, as mentioned above, the definition of successful aging in the scientific community is still controversial. The cumulative successful aging index that was developed [17] by simply adding the presence of the common determinants of the individuals may not accurately estimate the successful aging status. This methodology however, was based on a standard procedure described in the literature and previously used in other aging-associated definitions (i.e., frailty, healthy aging) [35, 36]. The absence of other attributes, such as physical function, dependence on others and self-management, may be considered as a limitation. However, the determinant of physical function was partially taken into account, through the inclusion of the frequency of physical activity.

### Conclusions

Elder residents of the continental Mani area enjoyed a better health status whereas elder islanders had higher level of successful aging. Lifestyle habits, like dietary, physical activities and socializing, seem to play an important role on successful aging. Public health authorities should focus on these determinants of healthy aging and promote socializing among elderly, for example by sharing food and related healthy traditions, going for outdoor activities or participating in social events organized by the local communities.
